# PD191 Global Disparities In Bariatric Surgery Reimbursement: A Comparative Analysis Of 23 Countries

**DOI:** 10.1017/S0266462324004136

**Published:** 2025-01-07

**Authors:** Birol Tibet, Amir Mustapha Sharaf, Guvenc Kockaya

## Abstract

**Introduction:**

Bariatric surgery is a weight loss procedure that supports significant and long-term weight loss for individuals with severe obesity by altering the digestive system. It improves health conditions associated with obesity and can be life changing. The purpose of the study was to examine the reimbursement criteria for bariatric surgery in certain countries.

**Methods:**

A detailed review was conducted on bariatric surgery reimbursement criteria across 23 countries, analyzing official policies and guidelines from databases and government websites. The study compared national data to highlight similarities and differences in these criteria, offering insights into the varying reimbursement approaches for bariatric surgery internationally.

**Results:**

The research compared global reimbursement policies for bariatric surgery, revealing that Australia, Belgium, Canada, Denmark, Egypt, France, Germany, Italy, Morocco, Russia, Saudi Arabia, Spain, Sweden, the Netherlands, Türkiye, and the UK support reimbursement for those with a body mass index over 40 kg/m^2^. However, Hungary, Nigeria, Libya, and Romania do not offer such reimbursements. This discrepancy highlights the diverse healthcare approaches to severe obesity treatment across countries. The absence of financial support in some regions indicates varying health priorities and economic constraints. These findings emphasize the need for harmonized global health policies, especially for obesity, which is a significant public health issue.

**Conclusions:**

The study highlights substantial disparities in bariatric surgery reimbursement criteria across countries. These differences, despite the proven benefits of treating severe obesity and related health issues, suggest a need for more standardized reimbursement approaches. Future research should explore the specific conditions and requirements for reimbursement and how these policies impact access to and outcomes of bariatric surgery.

